# Behavioral studies in a new mouse model of hemochromatosis mutations and Alzheimer’s amyloidosis

**DOI:** 10.1002/alz.090123

**Published:** 2025-01-03

**Authors:** Elvis Acquah, Daniel Johnstone, Adrienne (Lz) Milward

**Affiliations:** ^1^ University of Utah, Salt Lake City, UT USA; ^2^ University of Newcastle, Callaghan, NSW Australia; ^3^ University of Wollongong, Wollongong, NSW Australia

## Abstract

**Background:**

Brain iron dyshomeostasis has been observed in behavioral deficits relevant to neurodegenerative diseases such as Alzheimer’s disease (AD), but it remains unclear whether it is a primary cause or an epiphenomenon of disease.

**Method:**

We assessed the effects of brain iron dyshomeostasis on spatial cognition and cognitive flexibility using the IntelliCage system, recognition memory using novel object recognition tasks and anxiety‐like behavior using the open field and elevated plus maze tests. We investigated these phenotypes in a *Hfe^‐/‐^xTfr2^mut^* mouse model of brain iron dyshomeostasis alone (Iron^dys^) or combined with an APP^swe^/PS1^∆E9^ model of Alzheimer’s Aβ amyloidosis (Aβ+Iron^dys^), compared with APP^swe^/PS1^∆E9^ mice with Aβ amyloidosis alone (Aβ) or wildtype controls.

**Result:**

Brain iron levels in the Iron^dys^ models were ∼1.5x higher than wildtype control levels at 3 months of age, increasing to ∼1.8x or more at older ages. Brain iron dyshomeostasis alone had no effect on spatial cognition, cognitive flexibility or recognition memory. It moderately accelerated AD‐related anxiety‐like behavior in males when combined with Aβ, with Aβ+Iron^dys^ males spending less time in exposed areas of the open field arena than Aβ males at age 6 months (*n* = 7‐13 mice/group/sex, *p*˂0.05). At this age, irrespective of Aβ status, both male and female mice with brain iron dyshomeostasis also covered less distance in the elevated plus maze (*n* = 6‐10 mice/group/sex, *p*˂0.0001), while males of this group demonstrated this phenotype in the open field (*n* = 7‐13 mice/group/sex, *p*˂0.01). This iron‐related effect on locomotor activity was diminished by 9 months of age in the open field, with only the male Iron^dys^ groups covering less distance than the Aβ groups (*n* = 7‐13 mice/group/sex, *p* = 0.02). Interestingly, at 9 months of age, there was a dramatic reduction in locomotor activity of both Aβ mice and wildtype controls in the elevated plus maze, suggesting brain iron dyshomeostasis drives a natural age‐related decline in locomotor activity.

**Conclusion:**

While there was generally no strong effect of brain iron dyshomeostasis on most of the AD‐related behavioral phenotypes investigated, there was striking impairment of locomotion, independent of AD‐related mutations and amyloidosis. This suggests movement impairment may be an earlier feature of brain iron dyshomeostasis than cognitive decline.

